# Supportive interventions to improve physiological and psychological health outcomes among patients undergoing cystectomy: a systematic review

**DOI:** 10.1186/s12894-018-0382-z

**Published:** 2018-08-24

**Authors:** Helen Quirk, Derek J. Rosario, Liam Bourke

**Affiliations:** 1 Centre for Sport and Exercise Science, Sheffield Hallam University, S10 2BP, Sheffield, UK; 2 Department of Oncology, University of Sheffield, Sheffield, UK; 3 Faculty of Health and Wellbeing, Sheffield Hallam University, Sheffield, UK

**Keywords:** Bladder cancer, Cystectomy, Supportive intervention, Systematic review

## Abstract

**Background:**

Our understanding of effective perioperative supportive interventions for patients undergoing cystectomy procedures and how these may affect short and long-term health outcomes is limited.

**Methods:**

Randomised controlled trials involving any non-surgical, perioperative interventions designed to support or improve the patient experience for patients undergoing cystectomy procedures were reviewed. Comparison groups included those exposed to usual clinical care or standard procedure. Studies were excluded if they involved surgical procedure only, involved bowel preparation only or involved an alternative therapy such as aromatherapy. Any short and long-term outcomes reflecting the patient experience or related urological health outcomes were considered.

**Results:**

Nineteen articles (representing 15 individual studies) were included for review. Heterogeneity in interventions and outcomes across studies meant meta-analyses were not possible. Participants were all patients with bladder cancer and interventions were delivered over different stages of the perioperative period. The overall quality of evidence and reporting was low and outcomes were predominantly measured in the short-term. However, the findings show potential for exercise therapy, pharmaceuticals, ERAS protocols, psychological/educational programmes, chewing gum and nutrition to benefit a broad range of physiological and psychological health outcomes.

**Conclusions:**

Supportive interventions to date have taken many different forms with a range of potentially meaningful physiological and psychological health outcomes for cystectomy patients. Questions remain as to what magnitude of short-term health improvements would lead to clinically relevant changes in the overall patient experience of surgery and long-term recovery.

**Electronic supplementary material:**

The online version of this article (10.1186/s12894-018-0382-z) contains supplementary material, which is available to authorized users.

## Background

Perioperative complications from cystectomy and urinary diversion can be short- and long-term, physiological and psychological [1]. Postoperative morbidity and complication rates can lead to long hospital stays [2] and high readmission rates [3]. Surviving patients can experience emotional, physical and social challenges and changes in quality of life (QOL) [1]. The range of perioperative complications associated with cystectomy procedures requires a multidisciplinary approach to preoperative supportive care and postoperative rehabilitation [4].

Perioperative interventions should support patients’ psychological health as much as physical health [5]. The optimal perioperative supportive interventions for cystectomy patients and associated health outcomes are currently uncertain. Evidence-based interventions have traditionally been non-standardised but have evolved into clinical pathways of care known as enhanced recovery after surgery (ERAS) protocols. ERAS protocols involve a series of perioperative care modifications and supportive interventions with the aim to achieve early recovery by maintaining preoperative organ function and reducing physiological stress response following surgery [6]. ERAS protocols after cystectomy have had a low adoption [7], yet have been found to shorten hospital stay [3] without an increase postoperative morbidity [8]. Our understanding of the active ingredients of such protocols and how these may affect the overall patient experience in the long-term is limited and previous comprehensive reviews have involved non-randomised observational studies only [9] . Further exploration of the available evidence using rigorous systematic review methodology is required to develop our understanding of how to promote clinically relevant health outcomes for cystectomy patients.

The aim of this review is to summarise the available evidence base for any supportive interventions designed to improve short and/or long-term physiological and psychological health outcomes among patients undergoing cystectomy. Reviewing the literature of the wide range of perioperative supportive interventions and their related health outcomes will advance our understanding of what works for patients undergoing cystectomy.

## Methods

A systematic review of the literature was performed in January 2018. Records were identified from MEDLINE, AMED, PsycInfo and EMBASE databases and the Cochrane collaboration. The search was limited to studies involving adult humans and published in the English language and not limited by date of publication. Literature search terms are available as supplementary material (see Additional file 1). Further searches were made for unpublished and grey literature. The http://www.clinicaltrials.gov website was searched for ongoing trials. The citation lists of included studies and previous systematic reviews were also checked to identify relevant studies.

Randomised controlled trials (RCTs) involving any non-surgical, perioperative interventions designed to support or improve the patient experience, including lifestyle, physical, medical and psychological treatments were considered for review. The intention was not to assess the effects of different forms of surgical diversion. Studies were eligible if they involved adults ≥18 years who were due to undergo or had undergone a cystectomy procedure and any method of urinary diversion. Supportive interventions could be implemented during diagnosis and treatment planning, the perioperative period, and during the length of hospital stay, follow-up and postoperative period. Interventions could be hospital-based or home-based. Comparison groups included those exposed to usual clinical care or standard procedure. Studies were excluded if they did not involve an intervention, or the intervention involved a surgical procedure only, bowel preparation only or an alternative therapy such as aromatherapy. Any outcomes reflecting the patient experience or related urological health outcomes were considered and could be physiological, psychological, behavioural and social.

### Data collection and analysis

#### Selection of studies

**Fig. 1:**
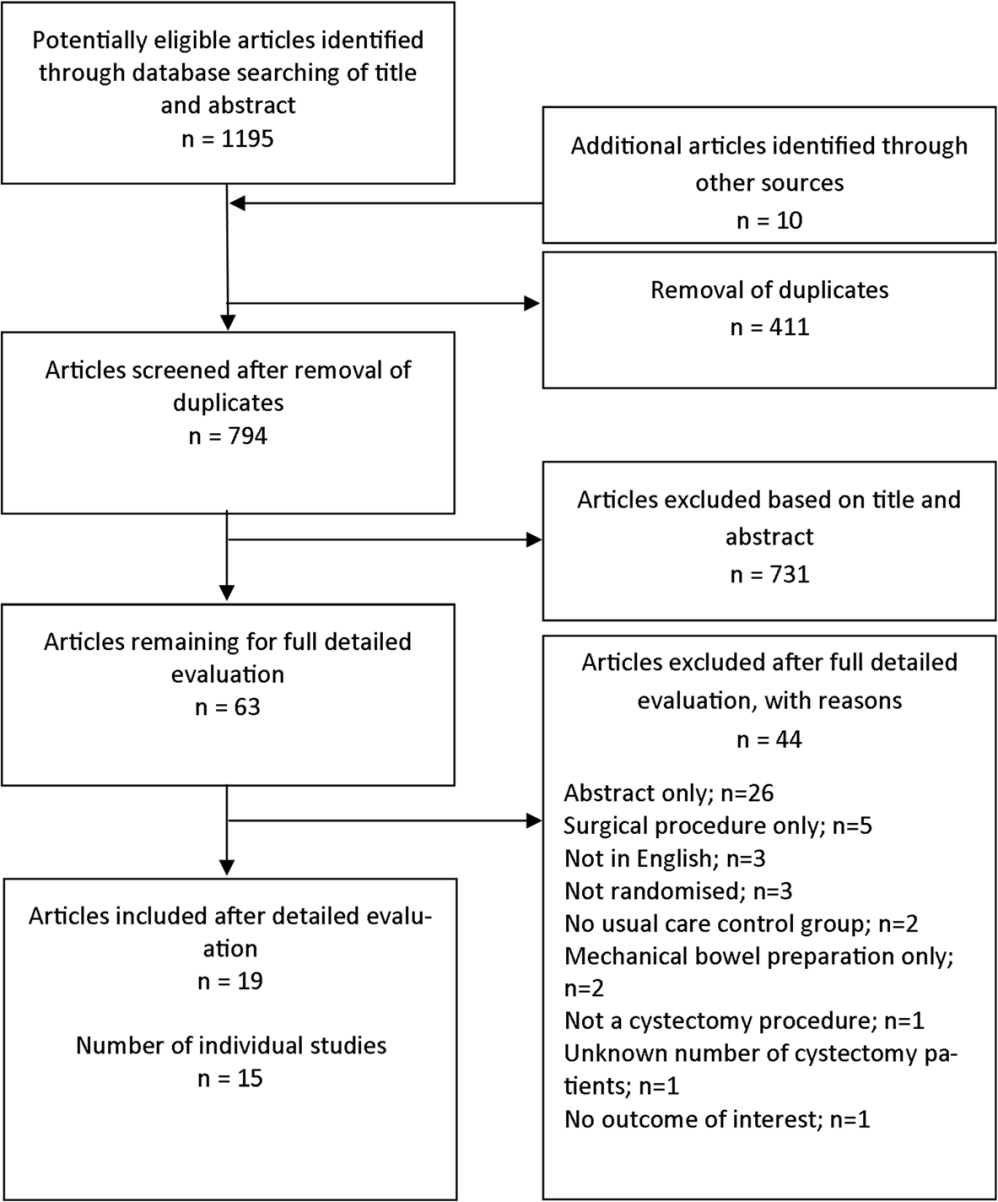
Flowchart describing the process of identifying relevant literature

Following de-duplication, titles and abstracts of identified records were screened by one reviewer (HQ) and 10% were selected at random and checked independently by a second reviewer (LB). The full texts of potentially eligible records were retrieved and screened independently by the two reviewers (HQ, LB). Multiple records of the same study were linked together in the process. The study selection process is described in the PRISMA flow diagram (Fig. 1).

#### Data extraction and management

The full text of each article was read by two reviewers independently (HQ, LB) and after piloting of extraction tables, relevant data were extracted. Any discrepancies in data extraction between the two reviewers were resolved by discussion. The authors of included studies were contacted via email for clarification of unclear study methods or data wherever insufficient details were reported.

#### Assessment of risk of bias in included studies

The risk of bias of each included study was assessed by two reviewers (HQ, LB) working independently using the recommended tool in the Cochrane Handbook for Systematic Reviews of Intervention [10]. Any disagreements were resolved by discussion.

#### Dealing with missing data

Missing data and dropout rates for each of the included studies were assessed. When possible, all data extracted were relevant to an intention-to-treat analysis, in which participants were analysed in the groups to which they were assigned.

#### Assessment of heterogeneity and sensitivity analyses

Statistical methods for assessing heterogeneity and sensitivity analyses were planned, depending on the availability of data.

#### Data synthesis and statistical analysis

Meta-analyses were planned for wherever there was more than one RCT reporting the same outcome. Where meta-analyses were not feasible, a narrative synthesis approach was used [11].

## Results

The search identified 63 articles meeting the inclusion criteria for full text screening (Fig. 1). In all, 44 articles were excluded and the reasons recorded. The remaining 19 articles (representing 15 individual studies) were included in the review. Studies were published between 1989 [12] and 2017 [13–15] and were conducted in ten different countries; one was UK-based [14] (see Table 1).

**Table 1:** Summary of study details and participant characteristics

Reference and country	Sample size			Participant characteristics						
				Age		Sex	Condition	Surgery procedure (as reported)	Urinary diversion type	Surgery type
	Total	INT	CONT	INT	CONT					
Ali et al., 1989 [12] Egypt	30	15	15	Mean 45.33 SD 5.9	Mean 45.86 SD 4.4	Male = 23 Female = 7	Bladder cancer	Urinary diversion	Not reported	Not reported
Banerjee et al., 2017 [14] UK	60	30	30	Mean 71.60 SD 6.80	Mean 72.5 SD 8.49	Male = 53 Female = 7	Bladder cancer	Radical cystectomy and urinary diversion	Not reported	Any surgical technique
Choi et al., 2011 [26] Korea	62	30	31	Mean 63.5 SD 4.5	Mean 64.5 SD 8.8	Not reported	Bladder cancer	Radical cystectomy and urinary diversion	Ileal conduit Orthotopic neobladder	Open and robot-assisted
Deibert et al., 2016 [28] USA	102	50	52	Not reported	Not reported	Male = 37 Female = 13	Bladder cancer	Radical cystectomy and urinary diversion	Ileal conduit Neobladder Pouch	Open and robot-assisted
Frees et al., 2017 [25] Canada	23	10	13	Mean 65.75 Range 49–86	Mean 70.40 Range 51–84	Male = 18 Female = 5	Bladder cancer	Radical cystectomy and urinary diversion	Ileal conduit Studer neobladder	Open and robot-assisted
Ghoneim & Hegazy, 2013 [22] Egypt	60	30	30	Mean 50.5 SD 11.2	Mean 49.4 SD 10.2	Male = 45 Female = 15	Bladder cancer	Radical cystectomy and urinary diversion	Not reported	Not reported
Jensen, Jensen et al., 2014, 2015, 2016, 2017 [13, 18–20] Denmark	107	65	64	Mean 68.5 SD 9.8	Mean 70.6 SD 9.2	Male = 79 Female = 28	Bladder cancer	Radical cystectomy	Ileal conduit Orthotopic neobladder Continent cutaneous reservoir	Open and robot-assisted
Karl et al., 2014 [24] Germany	101	62	39	Not reported	Not reported	Not reported	Bladder cancer	Radical cystectomy	Ileal conduit Orthotopic neobladder	Not reported
Lee et al., 2014 [16] USA	280	143	137	Mean 66 SD 10.9	Mean 64 SD 9.8	Male = 223 Female = 57	Bladder cancer	Radical cystectomy and urinary diversion	Orthotopic neobladder Continent cutaneous reservoir Noncontinent cutaneous reservoir	Open and robot-assisted
Månsson et al., 1997 [1] Sweden	57	24	26	Not reported	Not reported	Not reported	Bladder cancer	Radical cystectomy	Orthotopic neobladder	Not reported
Merandy et al., 2017 [15] USA	8	4	4	Median 74.5 IQR 73–81	Median 72 IQR 62–81.5	Male = 8 Female = 0	Bladder cancer	Radical cystectomy and urinary diversion	Orthotopic neobladder Incontinent conduit	Not reported
Mohamed et al., 2016 [23] Egypt	60	45 (15 per INT group)	15	Group 2 Mean 54.53 SD 8.56	Mean 47.80 SD 7.23	Male = 48 Female = 12	Bladder cancer	Radical cystectomy	Not reported	Not reported
				Group 3 Mean 54.20 SD 10.65						
				Group 4 Mean 53.33 SD 10.0						
Olaru et al., 2015 [17] Romania	20	10	10	Median 62.5	Median 62.0	Male = 20 Female = 0	Bladder cancer	Radical cystectomy and ileal urinary diversion	Orthotopic neobladder Bricker diversion	Not reported
Porserud et al., 2014 [21] Sweden	18	9	9	Mean 72 SD 5	Mean 72 SD 4	Male = 14 Female = 4	Bladder cancer	Radical cystectomy and urinary diversion	Ileal conduit	Open
Roth et al., 2013 [27] Vidal et al., 2016 [29] Switzerland	157	74	83	Median 67 Range 34–80	Median 66 Range 30–86	Male = 106 Female = 51	Bladder cancer	Radical cystectomy, extended pelvic lymph node dissection, and ileal diversion	Ileal conduit Ileal orthotopic bladder substitute Catheterisable pouch	Not reported

*CONT* Control, *INT* Intervention, *SD* standard deviation

### Participants

Table 1 provides a summary of participant characteristics. All studies involved patients with bladder cancer undergoing radical cystectomy. Sample sizes ranged from 8 [15] to 280 [16], with a total of 1145 participants across all studies. The average age of participants ranged from 45.3 years (mean) [12] to 74.5 years (median) [15]. Most studies included both sexes, except two studies that included males only [15, 17]. Other patient characteristics, though not reported consistently included BMI, ethnicity, comorbidities, smoking history, and socio-economic data.

### Interventions

See Table 2 for a summary of interventions included in this review.

**Table 2:** Summary of intervention details and length of follow-up

Intervention type	Author and date	Recruitment and setting	Perioperative stage and delivery	Intervention content	Intervention time, duration, frequency	Length of follow-up
Exercise therapy	Banerjee et al., 2017 [14]	Patients recruited from a single hospital. Supervised intervention setting.	Preoperative intervention delivered by exercise science staff	Short-term preoperative vigorous intensity aerobic interval exercise on a cycle ergometer using the Borg Ratings of Perceived Exertion (RPE) Scale to control intensity. 5–10 warm up against light resistance (50 W), patients aimed to perform 6 × 5 min intervals to a target perceived exertion of 13–15 (somewhat hard to hard equating to 70–85% predicted max heart rate based on 220-age, with 2.5 min interpolated active rest intervals against light resistance (50 W). Instructed to maintain a steady pedalling cadence of 50–60 rev min-1 during intervals, and the exercise programme was progressed gradually adding more load to the flywheel to maintain the target perceived exertion. Followed by cool down against low resistance (50 W).	5–10 warm up.6 × 5 min intervals with 2.5 min interpolated active rest intervals. Twice weekly over preoperative period until surgery (3–6 weeks). Minimum of six sessions performed.	Until discharge
	Jensen, Jensen et al., 2014 [18]	Patients recruited from a single hospital. Combined hospital and home-based intervention setting	Pre- and postoperative intervention delivered by physiotherapists	Preoperative standardised exercise training programme at home; step training on a step trainer and muscle strength and endurance exercises. Postoperative mobilisation and rehabilitation; instructions for getting out of bed, mobilisation and walking. Exercise-based rehabilitation in the hospital; respiratory and circulatory exercises, mobilisation, walking, supervised standardised progressive muscle strength and endurance training. Patients discharged with a home training exercise programme.	Preoperative 15 min step training and daily exercise programme consisting of six different exercises with individualised repetitions twice-daily. Postoperative mobilisation and exercise-based rehabilitation for 30 min twice-daily for the first seven postoperative days.	Day 35 and 4 months postoperatively
	Jensen, Petersen et al., 2015 [20]					
	Jensen, Laustsen et al., 2016 [19]					
	Porserud et al., 2014 [21]	Patients recruited from a single hospital. Combined hospital and home-based intervention setting	Postoperative intervention delivered by physiotherapists	Postoperative group exercise training programme in the hospital; lower body strength and endurance training; walking and strengthening exercises, balance training, mobility training and stretching exercises. Music was used as inspiration. Participants were also instructed to take walks at a self-selected pace.	45 min twice a week for 12 weeks. Walks at a self-selected pace, 3–5 days a week for at least 15 min.	14 weeks and 1 year postoperatively
Pharmaceutical	Ghoneim & Hegazy 2013 [22]	Recruitment setting not reported. Hospital based intervention	Preoperative intervention. Deliverer not reported	75 mg pregabalin orally.	2× day for 10 days prior to operation.	48 h postoperatively
	Lee et al., 2014 [16]	Patients recruited from multiple centres. Hospital based intervention	Pre- and postoperative intervention. Deliverer not reported	12 mg alvimopan before surgery and twice-daily doses postoperatively.	Single dose (12 mg) between 30 min and 5 h before surgery and twice-daily doses postoperatively until hospital discharge or a maximum of 7 days (15 in-hospital doses).	Until discharge and 30 days after discharge
	Mohamed et al., 2016 [23]	Patients recruited from single hospital. Hospital based intervention	Preoperative delivered by staff nurse	Group 2300 mg pregabalin orally 2 h preoperative Group 3300 mg pregabalin orally 2 h preoperative and 12 h thereafter Group 4600 mg pregabalin orally 2 h preoperative		24 h postoperatively
Fast-track/ERAS protocol	Frees et al., 2017 [25]	Patients recruited from single hospital. Hospital based intervention	Perioperative intervention. Deliverer not reported.	ERAS protocol (see original study for details).	Perioperative until discharge.	30 days postoperatively
	Karl et al., 2014 [24]	Recruitment setting not reported. Hospital based intervention	Perioperative intervention. Deliverer not reported	ERAS protocol (see original study for details).	Perioperative until discharge.	Day 3, day 7 postoperatively and until discharge
	Olaru et al., 2015 [17]	Patients recruited from a single hospital. Hospital based intervention	Perioperative intervention delivered by healthcare professionals	ERAS protocol (see original study for details).	Perioperative until discharge.	Until discharge
Psychological/educational	Ali et al., 1989 [12]	Patients recruited from a single hospital. Hospital based intervention	Preoperative intervention. Deliverer not reported	Single, preoperative psychoeducational session provided to the patient and a significant other. Included explanation of the surgical procedure, site and appearance of stoma, device to be used postoperatively, reasons for wearing a collection device, and a visit from another “ostomate” who is functioning well. Patients encouraged to express fears and anxieties regarding social aspects of living with a stoma, including clothing, changes in body image, sexuality, exercise, activity, and odour.	1 × 30–60 min session.	Until discharge (approx. 12 days postoperatively)
	Jensen, Kiesbye et al., 2017 [13]	Patients recruited from a single hospital. Combined hospital and home-based intervention setting	Pre- and postoperative intervention delivered by Urological Enteral Stoma Therapy Nurses	The education programme included basic skills to optimise the ability to perform independent stoma care. Patients encouraged to perform stoma care and change of appliance, both one-piece and two-piece system, at least twice at home providing them with training kits and appliances. The patient was educated about the urostomy and life with a urostomy related to the individual patient’s life and life style. Every patient had a follow up prior to surgery where the Urological Enteral Stoma Therapy Nurse observed self-care skills regarding stoma care and change of appliance.	1 x education programme under supervision, 2 x practice at home, 1 x self-demonstration under observation prior to surgery.	Day 35 and 4 months and 12 months postoperatively
	Mansson et al., 1997 [1]	Recruitment setting not reported. Home based intervention	Postoperative intervention. Deliverer not reported	Psychosocial programme including weekly counselling, in the patient’s home for 4 weeks, and thereafter by telephone. The discussion concerned consequences of the operation, practical and emotional problems, influences on mood and relations to partner and friends. The partner could be present at the interview.	Weekly counselling for 4 weeks then via telephone for 2 weeks.	3 months and 6 months postoperatively
	Merandy et al., 2017 [15]	Patients recruited from a single hospital. Hospital based intervention	Postoperative day 4, 5 or 6 delivered by trained nurse practitioners	Multimethod educational intervention was developed for each of the three different urinary diversions and included (a) a simplified medical illustration of participant-specific urinary diversion, (b) a step-by-step urinary diversion self-care instructional video, and (c) a pictorial Microsoft PowerPoint®. The content was driven by Bandura’s (1977) four sources of self-efficacy and were based on first-hand observed difficulties experienced by patients with a urinary diversion. The video, PowerPoint, illustrations, and surveys were administered at the bedside by one of the investigators using a tablet computer. The intervention was enhanced by professional demonstration, followed by a chance for return demonstration.	1 × 1 h in duration, with an optional 30 min for participant questions	Immediately after intervention
Chewing gum	Choi et al., 2011 [26]	Patients recruited from a single hospital. Hospital based intervention	Postoperative intervention delivered by study investigators	Sugar-free chewing gum.	30 min chewing three times daily at 10 am, 3 pm and 8 pm until first flatus.	Discharge. Short term complications within 30 days
Nutritional	Deibert et al., 2016 [28]	Patients recruited from 2 hospital centres. Hospital based intervention.	Postoperative intervention. Deliverer not reported	Clear liquid diet on postoperative day 1 and access to a full regular diet from postoperative day 2 and beyond.	Postoperative until discharge	90 days postoperatively
	Roth et al., 2013 [27]	Patients recruited from a single hospital. Hospital based intervention	Postoperative intervention delivered by hospital ward staff	Total parenteral nutrition (TPN). Nutriflex special; a solution with a total energy of 1240 kcal/1000 ml and containing polyamino acids, glucose, and electrolytes. An additional 30 IU Actrapid HM and 1875 IU heparin per 24 h were added to the TPN solution.	Administered continuously for 5 days starting on postoperative day 1.	1, 3, 7, 12 days postoperatively and complications up to 30 days postoperatively
	Vidal et al., 2016 [29]					3, 6, 12, 18, 24, 30 and 36 months postoperatively

#### Type

Intervention types included; exercise therapy [14, 18–21], pharmaceutical [16, 22, 23], ERAS protocol [17, 24, 25], psychological/educational [1, 12, 13, 15], chewing gum [26], and nutritional [27–29]. Interventions were delivered by exercise science staff [14], physiotherapists [18–21], Urological Enteral Stoma Therapy Nurses [13], trained nurse practitioners [15], hospital ward staff [27], and staff nurses [23], healthcare professionals [17] and study investigator [26]. Seven did not report who delivered the intervention [1, 12, 16, 22, 24, 25, 28]. Treatments to control group patients were determined by the standard procedure at the local hospital which may have involved some ERAS items [18–20, 25] and were not consistent across studies.

#### Recruitment and intervention setting

The majority of studies recruited participants via a single hospital urology department, two studies recruited across multiple centres [16, 28] and three did not report recruitment setting [1, 22, 24]. Intervention settings were hospital based [12, 15–17, 22–29], hospital and home based [13, 18–21], home-based [1] or supervised exercise setting [14].

#### Time, duration and frequency

Studies varied in time of intervention delivery; preoperative, postoperative or perioperative (see Table 2). Duration of intervention varied from 30 to 60 min for a single educational intervention [12, 15] to 12 weeks for the physical exercise intervention [21]. Six studies did not have standardised intervention duration; Banerjee et al.’s [14] exercise intervention took place preoperatively until surgery, Choi et al.’s [26] chewing gum intervention continued until first flatus, Deibert et al.’s [28] dietary intervention was postoperative until discharge, and those studies implementing ERAS protocols took place over the perioperative period until discharge [17, 24, 25]. Frequency of intervention administration differed depending on the intervention type (see Table 2).

### Measurements

Methods of measuring outcomes varied across studies, making direct comparisons between studies difficult. Hospital records were used to measure length of stay (LOS) and readmission rate. Hospital measurements were used to assess functions such as bowel function and flatus, food tolerance and mobilisation. Complications were assessed using the standardised Clavien-Dindo classification system [14, 17, 20, 25–28] or via hospital reports. Symptoms (e.g., pain, fatigue, vomiting) tended to be self-reported using patient questionnaires. Three studies [18, 24, 25] used the validated European Organisation for Research and Treatment of Cancer (EORTC) [30] to assess quality of life (QOL) and in-patient satisfaction. Three studies used a visual analogue scale (VAS) to measure pain intensity [22, 23, 28], one study used Sickness Impact Profile (SIP) to measure sickness-related dysfunction and postoperative adjustment [1], two studies used the Short Form health survey (SF-36 and SF-12) to evaluate health-related QOL [21, 29], one used the Functional Assessment of Cancer Therapy- Bladder Cancer (FACT-BL) questionnaire to measure QOL [25] and one used the State-Trait Anxiety Inventory (STAI) to measure state anxiety [12]. Self-care was measured using the Urostomy Education Scale (UES) [13]. Self-efficacy was measured using the six-item Self-Efficacy to Manage Chronic Disease (SES6G) scale [15].

Outcome measurement (length of follow-up) tended to be short term (up to 30 days postoperatively) in the majority of articles reviewed (*n* = 11), and ranged between 24 h postoperatively [23] to a median of 50 months after surgery (IQR 21–62 months) [29] (See Table 2).

### Effect of interventions

The outcomes used to measure the effect of interventions are summarised in Table 3. Differences in definitions and measurements of outcomes across studies meant that meta-analyses were not possible.

**Table 3:**
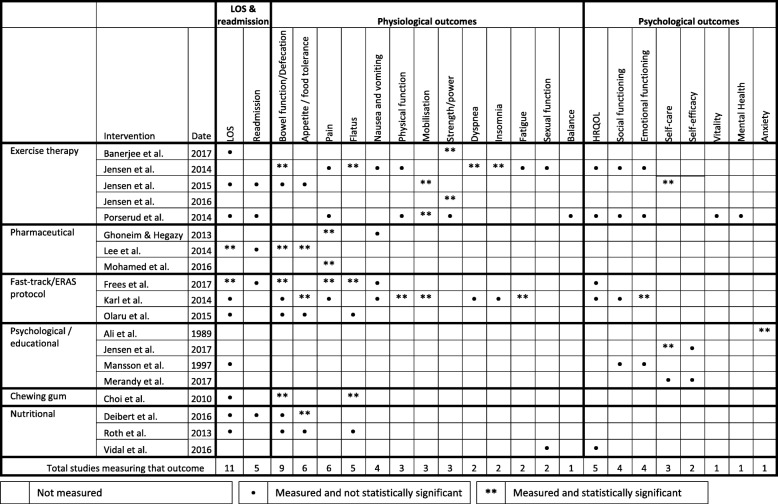
Summary of outcomes measured and statistically significant findings

#### Length of stay and readmission

Length of stay (LOS) was reported in 11 articles [1, 14, 16, 17, 20, 21, 24–28]. The most common definition of LOS was total hospital stay duration in days. Two studies defined it as postoperative days (from surgery until discharge) [1, 16]. Median LOS ranged from 7 [14] to 21 days [1]. Frees et al. [25] and Lee et al. [16] found a significant difference in LOS between intervention and control groups. Frees et al. found LOS was significantly shorter in the patients receiving ERAS protocol compared to standard procedure (mean 6.1 days vs. 7.39 days; *p* = 0.020). Lee et al. found mean LOS was significantly shorter in patients given alvimopan compared to placebo controls (alvimopan, 7.44 days; control 10.07 days; *p* < 0.01).

Frequency of readmission to hospital after discharge was measured as an outcome in five studies [16, 20, 21, 25, 28]. No study reported significant results for readmission rates after supportive intervention compared to controls.

#### Physiological adjustment after surgery

##### *Bowel function and flatus*

Nine studies measured bowel function [18, 20, 27, 28], also defined as time to first defecation or bowel movement [17, 25, 26], constipation [24] and lower gastrointestinal function [16]. Statistically significant reductions in average time until first bowel movement were found in four studies after the intervention; ERAS protocol [25], chewing gum [26], physical exercise [18] and alvimopan [16]. Time to first flatus was measured in five studies [17, 18, 25–27] and three found statistically significant reductions in time after ERAS protocol [25], chewing gum [26] and physical exercise [18]. Frees et al. [25] found significant reduction in time to first flatulence in the ERAS group compared to the standard procedure controls (2.5 days compared to 3.62 days) (*p* = 0.011).

##### *Food tolerance*

Six studies measured food tolerance, defined at nutritional intake [20], appetite loss [24], gastrointestinal recovery/tolerance of solid food [16], early feeding [17] and resumption of full diet [27]. Deibert et al. [28] found time to full diet tolerance was the same in both early diet and control arms, respectively (5.84 days vs 6.71 days, *p* = 0.27). Lee et al. [16] found mean time to gastrointestinal recovery was 1.3 days shorter for the alvimopan group (5.5 days) compared with the placebo control group (6.8 days; 95% CI, 1.4 to 2.3; *p* < 0.0001). Karl et al. [24] found that the amount of food consumed in relation to the amount of food offered on postoperative day 3 was significantly higher in the ERAS group compared to standard procedure controls (*p* = 0.02).

##### Nausea and vomiting

Four studies measured vomiting [22], nausea [25] or both [18, 24] and none reported any significant differences between intervention and control groups after the intervention.

##### Pain

Six studies measured pain [18, 21–25]. Three studies reported statistically significant pain outcomes. Ghoneim and Hegazy [22] found VAS score to be significantly lower postoperatively until 32 h in the intervention group receiving preoperative pregabalin compared to the control group (*p* < 0.05), but found no significant difference 32–48 h postoperatively. Mohamed et al. [23] found a significant reduction in VAS score in intervention groups who received preoperative pregabalin in comparison with the control group immediately after surgery, and 2 h postoperatively (*p* < 0.05). Frees et al. [25] found ERAS patients reported a reduction in VAS score every day after surgery until day 7 compared to patients undergoing standard procedure. This difference reached statistical significance on the day of surgery (*p* = 0.017) and from postoperative days 2 (*p* = 0.014) to 4 (*p* = 0.039), where pain intensity was nearly doubled for patients who received standard procedure.

##### Fatigue

Two studies measured fatigue using the EORTC symptom scale [18, 24]. Jensen et al. [18] found the control group (no physical exercise intervention) demonstrated a clinically relevant reduction in fatigue symptoms at 4 months follow-up that was not statistically significant. Karl et al. [24] reported significant differences in fatigue scores between the ERAS and control group at day 7 (*p* = 0.014) and discharge (*p* = 0.003), but did not report the group data.

##### Mobilisation, strength/power and balance

Three studies measured mobilisation [20, 21, 24], defined as the distance walked during the first seven postoperative days [20], mobilisation and walking distance [24] and distance walked in the 6 min walk test [21]. Jensen et al. [20] reported significantly longer average walking distance in the intervention group after the physical exercise intervention (4806 m walked; 95% CI, 4075 to 5536 m), compared to the control group (2906 m walked; 95% CI, 2408 to 3404 m; *p* < 0.001). Karl et al. [24] reported that patients in the ERAS group covered significantly greater walking distances by postoperative day 3 compared to controls (*p* = 0.039). Porserud et al. [20, 21] found that after the 12 week exercise training period, both the intervention and the control group patients had increased the distance walked (*p* = 0.043 and *p* = 0.012, respectively), but the increase was higher among the intervention group (*p* = 0.013) who had exercised postoperatively. One year later, the exercise group continued to have increased walking distance compared to controls (*p* = 0.010).

The three studies using exercise therapy measured strength or power. Jensen et al. [19] measured strength as muscle leg power (W/kg) using a leg extensor power test and found that the prehabilitation physical exercise programme led to a significant improvement in muscle power in the intervention group of 0.35 W/kg (95% CI, 0.12 to 0.54) at time for surgery compared to baseline (*p* < 0.002) with a significant difference between intervention and control group. Banerjee et al. [14] implemented a short-term preoperative vigorous intensity aerobic interval exercise programme on a cycle ergometer and showed that after 3–6 weeks of training, statistically significant differences in peak power output (W) were found between the exercise group (148 ± 41; 95% CI, 132 to 165) compared to non-exercising controls (129 ± 44; 95% CI, 111 to 147; *p* < 0.001) [14]. Porserud et al. [21] measured lower body strength using a 30-s chair stand test and found no significant differences between the intervention and control group. Porserud et al. also measured balance by asking patients to walk two laps in a figure of eight drawn on the floor, with a walking aid if necessary and found no significant differences between intervention and control group post-intervention or 1 year later [20].

##### Physical function

Three studies measured physical function, two using the EORTC-QLQ-30 [18, 24] and one using the SF-36 [21]. No statistical differences were found, except for Karl et al.’s [24] study, which found statistically higher physical functioning scores on postoperative day 3 for patients in the ERAS group.

##### Dyspnoea

Dyspnoea was measured in two studies using the EORTC-QLQ-30 [18, 24]. Jensen et al. [18] found a 10% significant decrease in symptoms of dyspnoea in the intervention group (physical exercise rehabilitation) compared with the control group at 4 month follow-up. Karl et al. [24] reported no significant differences between intervention and control group after the ERAS protocol.

##### Insomnia

Insomnia was measured in two studies using the EORTC-QLQ-30 [18, 24] and no significant differences between intervention and control groups were found after the intervention.

##### Sexual function

Two studies measured sexual function [18, 29]. Jensen et al. [18] found an improvement of 7% in sexual interest and activity in the control group 4 months after the intervention, which they described as clinically relevant though it was not statistically significant. Vidal et al. [29] measured sexual function as a long-term follow-up to the TPN nutritional intervention described by Roth et al. [27] and found no statistically significant differences between intervention and control group at 0, 3, 12 and 24 month follow-ups.

#### Psychological adjustment after surgery

##### Social and emotional functioning

Four studies measured social and emotional functioning using EORTC-QLQ-30 [18, 24], the SF-36 [21] and the SIP questionnaire [1]. No study found statistically significant differences between intervention and control groups after the intervention except Karl et al. [24] who found a stable emotional functioning score during hospitalisation in the control group and continuous improvement in emotional functioning until discharge in patients exposed to the ERAS protocol (no data reported) [24].

##### Health related quality of life

Five studies measured QOL, one using the FACT-BL [25], two using global health-related QOL from the EORTC-QLQ-30 and functional subscales [18, 24] and two using the SF-12 or 36 [21, 29]. Porserud et al. [21] found no statistically significant differences between intervention and control group in the QOL domains. Jensen et al. [18] found the physical rehabilitation intervention group demonstrated a clinically relevant decrease compared to the control group on role function and cognitive function at the 4 month follow-up, although differences were not statistically significant. Frees et al. [25] and Vidal et al. [29] found no statistically significant differences between intervention and control groups in QOL scores.

##### Self-care and self-efficacy

Three studies measured self-care [13, 15, 20] and two measured self-efficacy [13, 15] as outcomes of the intervention. Jensen et al. [20] found the ability to independently perform personal activities of daily living was significantly reduced by 1 day in the intervention group after pre-and postoperative physical exercise intervention compared to controls (3 days vs 4 days; *p* ≤ 0.05). Jensen et al. [13] found no statistical significant difference (*p* = 0.35) in mean self-efficacy score between treatment groups on admission to surgery. However, a significant increase in the total stoma self-care score of 2.7 points (95% CI, 0.9 to 4.5) was found in the intervention group compared to the standard procedure group at postoperative day 35, and differences continued at day 120 (4.3 95% CI, 2.1 to 6.5) and 365 (5.1 95% CI, 2.3 to 7.8). Merandy et al. [15] found that the single preoperative educational intervention was not associated with self-care independence scores (*p* = 0.4286) and brought about no significant change in self-care or self-efficacy scores.

##### Other outcomes

Other outcome measures explored in isolation included vitality and mental health [21] and anxiety [12]. Porserud et al. [21] found no significant differences between intervention and control group in vitality and mental health scores as measured by the SF-36. Ali and Khalil [12] found patients who received psychoeducational preparation prior to surgery showed less state anxiety on the third day postoperatively than the control group (*p* < 0.00 [sic]) and before discharge (*p* < 0.00 [sic]) compared to controls. Through a qualitative analysis, Ali and Khalil [12] also found that patients fears and worries before surgery concerned i) cancer, ii) mutilation and body image distortion, and iii) impact on social/marital relationships.

#### Complications

Eleven studies reported complications associated with the surgical procedures, seven using the standardised Clavien-Dindo classification system [14, 17, 20, 25–28] (See Additional file 2). Generally, interventions were not found to substantially increase the normal complication rate, with the exception of one study that was terminated prematurely due to high gastrointestinal complications in patients exposed to total parenteral nutrition (TPN) for 5 days postoperatively [27].

#### Adherence and fidelity

Adherence to the intervention was reported in eight articles. Table 4 gives a summary of the adherence reported in each of the articles under review. Eleven articles did not report adherence to the intervention. Fidelity of the intervention delivery was not reported in any article.

**Table 4:** Adherence to the intervention

Paper	Adherence
Ali et al., 1989 [12]	Not reported
Banerjee et al., 2017 [14]	The median number of supervised exercise sessions attended by patients in the exercise arm was 8 (range 1–10) over a preoperative period of 3–6 weeks. The average number of aerobic intervals achieved in the first week of exercise was 5.5 (range 3.5–6.0), whereas all patients were achieving six intervals per session in the fourth week.
Choi et al., 2011 [26]	Not reported
Deibert et al. 2016 [28]	Not reported
Frees et al., 2017 [25]	Not reported
Ghoneim & Hegazy, 2013 [22]	100% adherence to pregabalin
Jensen et al., 2014 [18]	A total of 66% (95% confidence interval (CI) 51; 78) adhered more than 75% of the recommended progressive standardised exercise program.
Jensen et al., 2016 [19]	A total of 66% (95% confidence interval (CI) 51; 78) adhered more than 75% of the recommended progressive standardised exercise program.
Jensen et al., 2015 [20]	A total of 66% (95% confidence interval (CI) 51; 78) adhered more than 75% of the recommended progressive standardised exercise program.
Jensen et al., 2017 [13]	Not reported
Karl et al., 2014 [24]	Not reported
Lee et al., 2014 [16]	119 out of 143 (83%) patients completed the alvimopan
Mansson et al., 1997 [1]	Not reported
Merandy et al., 2017 [15]	Not reported
Mohamed et al., 2016 [23]	Not reported
Olaru et al., 2015 [17]	Counselling and education was implemented in 90% of patients
Porserud et al., 2014 [21]	Participants attended a median of 76% (range 67–95%) of the group exercise training sessions and patients self-reported daily walks on 87% (56–100%) of the days during the 12-week period, averaging 3.5 h (2–11.5%) per week
Roth et al., 2013 [27]	Not reported
Vidal et al., 2016 [29]	Not reported

### Risk of bias

**Fig. 2:**
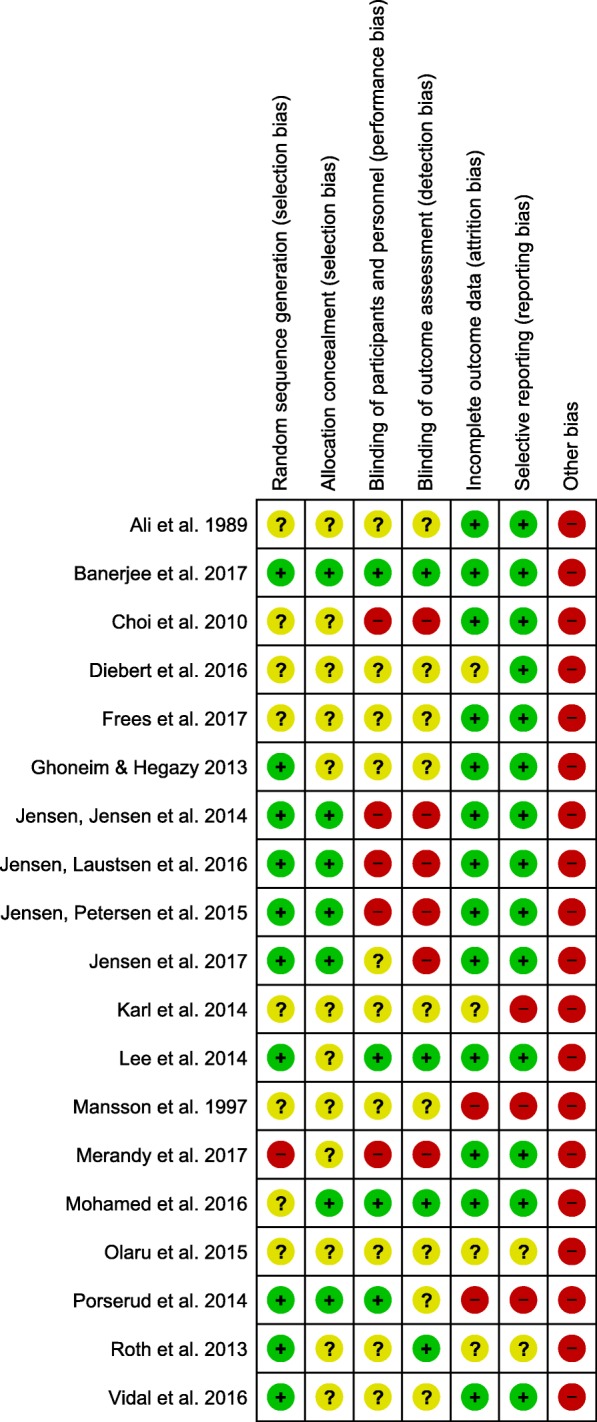
Risk of bias summary table

Figure 2 shows the risk of bias summary table for the studies included. The standard of reporting was generally low, with many articles omitting Consolidated Standards of Reporting Trials (CONSORT) details [31]. Low reporting quality meant the majority of studies were judged to have unclear risk of bias on at least one domain. All studies were described as having randomised designs, but only ten articles reported the randomisation procedure (e.g., web-based block randomisation [18]). In eight articles, it was unclear how participants were randomised. One study was described as randomised but did not describe a true randomisation procedure, therefore considered high risk of bias [15]. Seven studies were rated low risk for ‘selection bias’, because they referred to allocation concealment in their reporting of the randomisation procedure [13, 18–21, 23]. Studies tended to be rated as unclear or high risk for ‘performance bias’ and ‘detection bias’ because it was unclear whether patients, study personnel or outcome assessors were blind to the treatment group. Double-blind RCTs are difficult, if not impossible for many non-pharmaceutical intervention studies, exposing most of the studies to performance bias. Two studies included in the review were described as double-blind [16, 23]. All studies were judged to be at high risk of some ‘other bias’. This included, use of a single centre [12], different surgical and treatment procedures across different sites [16], LOS being influenced by hospital discharge rules (rather than health outcomes) [26], small sample sizes [1, 12, 17, 21, 22, 26], change over time in surgical procedure [18–20], intervention and control group patients being treated on the same hospital ward [18–20], use of male patients only [17], not recruiting the target sample size [21, 28] and premature termination of the study [27, 29].

### Heterogeneity and sensitivity analyses

Differences in the included studies, particularly in types of interventions, definitions of outcomes and tools used to measure outcomes meant sensitivity analyses could not be conducted and heterogeneity could not be assessed statistically.

## Discussion

Supportive interventions for cystectomy patients have included exercise therapy, pharmaceuticals, ERAS protocols, psychological/educational programmes, chewing gum and nutrition delivered at various stages over the perioperative period. It is difficult to make clear recommendations for clinical practice, especially for potential long-term benefits to patient health, but this review can offer suggestions for potential short-term benefits of interventions.

Review findings suggest that integrating exercise therapy into the pre- or postoperative care of cystectomy patients could have clinically important benefits for bowel function, physical function, strength/power, mobilisation and QOL but is not always feasible for patients. The findings align with other reviews demonstrating the positive effects of exercise for bladder cancer patients [32]. Exercise can be challenging for cancer patients and requires careful consideration with respect to patient age and comorbidities [18, 33]. Research exploring the optimal type of exercise therapy would be informative, as intensive exercise may not always be appropriate [21] or accessible [14] for patients undergoing cystectomy.

Cystectomy patients may benefit from pharmaceutical intervention for pain relief and physical function in the immediate postoperative period, which is likely to have a positive impact on length of hospital stay, QOL, the patient experience and healthcare costs. However, the effect on pain management might be short-lived and side-effects such as the sedative effect of pregabalin should be considered [22, 23].

Only three of the included studies used ERAS protocols [17, 24, 25], supporting the observation that the adoption of ERAS protocols in urological procedures to date has been low [6]. The findings suggest that ERAS protocols have the potential to offer widest range of benefits for cystectomy patients. However, it is hard to identify what actually works within each context and the quality and quantity of the evidence needs improvement. Tyson and Chang [9] systematically reviewed 13 studies comparing ERAS after cystectomy versus standard care with a meta-analysis of effectiveness. ERAS protocols were investigated within observational studies only and were found to reduce the LOS, time-to-bowel function, and rate of complications after cystectomy, but the pooled estimates were biased in favour of ERAS and each perioperative pathway was different within each study [9]. If ERAS protocols are to be adopted, then high-quality multicentre studies are needed to accumulate evidence supporting the short and long-term impact of their use.

The findings demonstrate that psychologically-supportive and educational interventions are less common than physical or medical interventions, but could reduce postoperative anxiety and promote postoperative adjustment, self-care and coping in cystectomy patients. Such outcomes are likely to benefit QOL and positive adjustments with clinical relevance [13], but are likely to require a longer and more individualised approach than those implemented in the studies included in this review. The findings are consistent with a previous systematic review of exercise and psychosocial rehabilitation interventions to improve health-related outcomes in patients with bladder cancer undergoing radical cystectomy, which found limited evidence for beneficial effects of psychosocial interventions [32]. Given that poor preoperative mental health has been associated with complications after cystectomy [34] and postoperative problems can have a significant impact on QOL [5], assessing perioperative psychological health status could help identify those patients who may be in need of extra support. Further research is required to explore the best approach to provision of psychological support for patients to ensure that patients are not only surviving, but surviving well.

Asking cystectomy patients to chew gum postoperatively may have benefits for bowel function and is unlikely to have any adverse effects. The early introduction of diet was feasible and safe, but TPN was associated with an increased rate of infectious complications, impaired bowel function, as well as higher costs [27].

Some level of bias was present in all studies included in this review, with most of the uncertainty in judging bias coming from lack of clarity of randomisation and blinding procedures. Methodological details were underreported and future publications should adequately report high quality research. No study reported fidelity of intervention delivery meaning it was unclear whether the treatment was delivered as intended. Additionally, the surgical procedure, including form of urinary diversion to control group patients varied across studies (see Table 1), introducing potentially confounding factors. This makes it difficult to show whether any health benefits were related to the supportive intervention or to determine the optimal ‘dosage’ or exposure to the intervention required to bring about health benefits. Many of the studies lacked statistical power due to small sample sizes meaning statistical significance should be interpreted with caution.

### Recommendations for future research

Implications for clinical practice have been difficult to make, suggesting that future research should explore the clinical relevance of the outcomes found in research studies. Maintenance data through longer follow-ups are essential to explore i) long-term complications and readmissions and ii) whether short-term health outcomes are sustained over time. Adequately powered clinical trials are required to explore the long-term effects of physical prehabilitation and rehabilitation for cystectomy survivors. More research exploring psychologically-supportive interventions would be informative because the current findings highlight that psychological and behavioural outcomes (e.g., self-care behaviour and behaviour change) are scarcely studied and poorly understood. Standards of reporting must be improved, including details of fidelity and adherence.

## Conclusions

This review provides a broad overview of the non-surgical supportive interventions available to help optimise the health outcomes of patients undergoing cystectomy procedures. It has shown that supportive interventions have taken many different forms with a range of potentially meaningful physiological and psychological health outcomes for patients in the short and long term after surgery. Questions remain as to what magnitude of improvements in the physiological and psychological health outcomes reported would lead to actual changes in the patient experience of surgery and recovery. Whilst this review can offer suggestions for potential benefits of interventions, clarification is required to understand what forms of support are most effective in improving the long-term quality of life of cystectomy patients.

## Additional files

Additional file 1: Search terms - Literature search terms for the electronic database search used in this review. (DOCX 22 kb)

Additional file 2: Summary of complications - Summary of reported complications for each study included in this review. (DOCX 30 kb)

## Abbreviations

CONSORT: Consolidated Standards of Reporting Trials
EORTC-QLQ-30: European Organisation for Research and Treatment of Cancer - Quality of life of cancer patients
ERAS: Enhanced recovery after survey
FACT-BL: Functional Assessment of Cancer Therapy- Bladder Cancer
LOS: Length of stay
QOL: Quality of life
RCT: Randomised controlled trial
SES6G: Self-Efficacy to Manage Chronic Disease scale
SF-36 and SF-12: Short Form health survey
SIP: Sickness Impact Profile
STAI: State-Trait Anxiety Inventory
UES: Urostomy Education Scale
VAS: Visual analogue scale
