# Supplementary material for: Supportive interventions to improve physiological and psychological health outcomes among patients undergoing cystectomy: a systematic review
Source: BMC Urol. 2018 Aug 24;18:71. doi: 10.1186/s12894-018-0382-z (PMC6109292; doi:10.1186/s12894-018-0382-z)
Supplement: Supplementary file 1 — Search terms - Literature search terms for the electronic database search used in this review. (DOCX 22 kb) [file 12894_2018_382_MOESM1_ESM.docx]

Supportive interventions to improve physiological and psychological health outcomes among patients undergoing cystectomy: A systematic review

Helen Quirk; Derek James Rosario; Liam Bourke

BMC Urology

**Additional File 1**

Search terms

|  | MEDLINE, EMBASE, PsycINFO, AMED, Cochrane | Search |
| --- | --- | --- |
| Condition of interest | 1] cystectomy OR cystourethrectomy OR cystoprostatectomy OR "anterior extenteration" OR "nerve sparing" OR "function sparing" OR urostom* OR "urinary diversion" OR "ileal conduit" OR "colonic conduit" OR "bladder reconstruction" OR "orthotopic neobladder" OR "ileal neobladder" OR "urinary tract reconstruction" "continent diversion" OR ureterosigmoidostomy OR "cutaneous ureterostomy" OR "cutaneous diversion" | Title and abstract |
| Intervention of interest | 2] intervention OR program* OR educat* OR "patient education" OR counsel* OR psycho* OR psychosocial OR "psychosocial intervention" OR manag* OR "self-mang*" OR support* OR "enhanced recovery" OR ERAS OR "recovery program*" OR "accelerated recovery" OR postoperat* OR perioperat* OR preoperat* OR rehabilit* OR multimodal OR "fast track" OR "fast-track" OR "decision aid" | Title and abstract |
| Study design | 3] random* OR "randomised controlled trial" OR "randomized controlled trial" OR RCT OR controlled OR "clinical trial*" OR "controlled clinical trial" | Title and abstract |
| Search combination | 1 AND 2 AND 3 |  |
