# Supplementary material for: Supportive interventions to improve physiological and psychological health outcomes among patients undergoing cystectomy: a systematic review
Source: BMC Urol. 2018 Aug 24;18:71. doi: 10.1186/s12894-018-0382-z (PMC6109292; doi:10.1186/s12894-018-0382-z)
Supplement: Supplementary file 2 — Summary of complications - Summary of reported complications for each study included in this review. (DOCX 30 kb) [file 12894_2018_382_MOESM2_ESM.docx]

Supportive interventions to improve physiological and psychological health outcomes among patients undergoing cystectomy: A systematic review

Helen Quirk; Derek James Rosario; Liam Bourke

BMC Urology

**Additional File 2**

Summary of complications

| **Reference** | **Complication definition** | **System** | **Classification** | **Complication description** | **Complication incidence** | | | |
| --- | --- | --- | --- | --- | --- | --- | --- | --- |
|  |  |  |  |  | | **Total n** | **INT n** | **CONT n** |
| Banerjee et al., 2017 | Post-surgical complications | Clavien–Dindo | Grade ≥ 1 | Not reported | |  | 4 (15%) | 10 (36%) |
|  |  |  | Grade ≥ 3 | Not reported | |  | 1 (4%) | 4 (14%) |
|  |  |  | Other | Ileus  Pneumonia | |  | 6  3 | 7  2 |
| Choi et al., 2010 | Short term complications (within 30 days) and gastrointestinal complications | Clavien–Dindo | Grade 1 | Urethral anastomosis leaks | | 3 |  |  |
|  |  |  | Grade 2 | Paralytic ileus  Delirium  Acute renal failure | | 5  1  4 | -  -  - | -  -  - |
|  |  |  | Grade 3 | Arteriosclerosis obliterans in the foot  Myocardiac infarction  Ureterointestinal anastomosis leaks | | 1  1  3 | -  -  - | -  -  - |
|  |  |  | Other | Gastrointestinal complications | | 5 | 2 | 3 |
| Deibert et al., 2016 | Complication rate | Clavien-Dindo | Overall complication rate | Not reported | | 65 | 34 | 31 |
|  |  |  | Inpatient complication rate | Not reported | | 42 | 21 | 21 |
|  |  |  | Post-discharge complication rate | Not reported | | 35 | 18 | 17 |
|  |  |  | Grade 5 | Mortality | | 6 | 2 | 4 |
|  |  |  | Other | Frequency of ileus | | 34 | 13 | 21 |
| Frees et al., 2017 | Complications | Clavien-Dindo | Grade < 2 | Minor complications: delirium and cardiac event | | 2 | 2 | 0 |
|  |  |  | Grade > 2 | Severe complications | | 0 | 0 | 0 |
| Ghoneim & Hegazy, 2013 | Side-effects within 48 hours postoperatively | N/A | N/A | Sedation score at 4h (median, range)  Nausea  Vomiting  Dizziness  Blurring of vision  Pruritus | | -  13  1  3  3  1 | 2 (1-3)  6  0  2  4  0 | 1 (1-2)  7  1  1  3  1 |
| Jensen, Jensen et al., 2015 | Early complications occurring within 90 days postoperatively | Clavien–Dindo | Grade 0 | Not reported | | 43 | 20 | 23 |
|  |  |  | Grade 1 | Not reported | | 24 | 9 | 15 |
|  |  |  | Grade 2 | Not reported | | 14 | 9 | 5 |
|  |  |  | Grade 3 | Not reported | | 16 | 8 | 8 |
|  |  |  | Grade 4 | Not reported | | 3 | 1 | 2 |
|  |  |  | Grade 5 | Death | | 4 | 3 | 4 |
| Karl et al., 2014 | Postoperative assessment of complications | N/A | N/A | Hydronephrosis  Urinary tract infection  Antibiotics for urinary tract infection  Cardiovascular complications  Deep vein thrombosis  Lung emboli  Wound healing disorders  Paralytic ileus  Fever | | -  -  -  -  -  -  -  -  - | 10%  40%  35%  8%  0%  2%  15%  15%  26% | 13%  38%  33%  13%  8%  8%  38%  28%  54% |
| Lee et al., 2014 | Incidences of nausea, vomiting, abdominal bloating, and antiemetic use. Incidence of CV adverse events (AEs) within 30 days postoperatively | N/A | N/A | Mild TEAE*  Moderate TEAE  Severe TEAE  Hypokalaemia  Anaemia  Postoperative ileus  Hypocalcaemia  Insomnia  Hypomagnesaemia  Nausea  Pyrexia  Tachycardia  Pruritus  Patients with >1 cardiovascular event  of interest  Congestive heart failure  Cardiovascular death  Cerebrovascular accident  Myocardial infarction  Serious arrhythmia  Unstable angina | | 55  131  59  58  52  46  45  32  31  30  29  27  25  33  4  5  2  14  15  2 | 31  67  26  24  25  10  21  17  18  10  14  12  10  12  0  1  1  4  8  1 | 24  64  33  34  27  36  24  15  13  20  15  15  15  21  4  4  1  10  7  1 |
| Mohamed et al., 2016 | Side-effects within 24 hours postoperatively | N/A | N/A | Nausea/Vomiting  Pruritus  Headache  Dizziness  Visual abnormalities | | 8  3  5  12  3 | 2  0  5  11  3 | 6  3  0  1  0 |
| Olaru et al., 2015 | Postoperative complications | Clavien–Dindo | Minor (Grade <3) | Postoperative ileus  Nausea/vomiting  Urinary tract infection  Arrhythmias  Atelectasis/desaturations  Altered mental status  Deep vein thrombosis  Urine leak  Clostridium difficile enterocolitis  Pneumonia  Wound dehiscence  Fever of unknown origin | | 6  3  2  1  0  0  0  1  4  0  5  2 | 2  1  0  0  0  0  0  1  1  0  2  0 | 4  2  2  1  0  0  0  0  3  0  3  2 |
|  |  |  | Major (Grade 3-5) | Eviscerating  Acute renal failure  Cardiac ischemia  Death  Postoperative bleeding  Pulmonary thromboembolism  Mechanical bowel obstruction | | 2  0  0  0  0  0  0 | 0  0  0  0  0  0  0 | 2  0  0  0  0  0  0 |
| Roth et al., 2013 | Complications within 30 d postoperatively | Clavien–Dindo | Not reported | *Infectious*:  Urinary tract infection  Pyelonephritis  Urosepsis  Sepsis  Pelvic abscess  Pneumonia  Sigmoid diverticulitis  Fever of unknown origin  Wound infection  *Pulmonary*:  Acute respiratory distress syndrome  *Gastrointestinal*:  Ileus  Small bowel obstruction (surgical treatment)  Gastrointestinal bleeding  Duodenal/gastric ulcer  Pancreatitis  *Cardiac*:  Arrhythmia  Myocardial infarction  Congestive heart failure  *Genitourinary*:  Temporary renal failure requiring haemodialysis  Uretero-ileal obstruction  Urinary retention  Urinary leak  *Bleeding*:  Postoperative haematoma (surgical treatment)  *Thromboembolism*:  Deep vein thrombosis  Pulmonary embolism  Acute mesenteric ischaemia *Neurologic*:  Delirium/agitation  *Miscellaneous*:  Lymphocele  Peripheral arterial ischaemia  Wound dehiscence  Other rare complications | | 3  6  4  3  2  7  2  7  6  2  20  2  1  1  1  3  3  2  1  2  2  3  1  3  3  2  2  13  1  3  5 | 2  4  4  3  2  4  2  5  4  2  12  2  -  1  1  -  2  -  1  2  1  2  1  2  1  1  1  8  1  2  2 | 1  2  -  -  -  3  -  2  2  -  8  -  1  -  -  3  1  2  -  -  1  1  -  1  2  1  1  5  -  1  3 |

*TEAE = treatment-emergent adverse events
